# Confinement dependence of electro-catalysts for hydrogen evolution from water splitting

**DOI:** 10.3762/bjnano.5.21

**Published:** 2014-02-24

**Authors:** Mikaela Lindgren, Itai Panas

**Affiliations:** 1 Department of Chemical and Biological Engineering, Chalmers University of Technology, S-412 96 Gothenburg, Sweden

**Keywords:** confinement, corrosion, DFT, electro-catalysis, hydrogen evolution

## Abstract

Density functional theory is utilized to articulate a particular generic deconstruction of the electrode/electro-catalyst assembly for the cathode process during water splitting. A computational model was designed to determine how alloying elements control the fraction of H_2_ released during zirconium oxidation by water relative to the amount of hydrogen picked up by the corroding alloy. This model is utilized to determine the efficiencies of transition metals decorated with hydroxide interfaces in facilitating the electro-catalytic hydrogen evolution reaction. A computational strategy is developed to select an electro-catalyst for hydrogen evolution (HE), where the choice of a transition metal catalyst is guided by the confining environment. The latter may be recast into a nominal pressure experienced by the evolving H_2_ molecule. We arrived at a novel perspective on the uniqueness of oxide supported atomic Pt as a HE catalyst under ambient conditions.

## Introduction

Molecular hydrogen produced by water splitting constitutes the archetypical energy carrier in chemistry and is a main target process for the future harvesting of solar energy. Today, water splitting represents large economical values, i.e., it comprises a significant fraction of the total industrial electric energy consumption in the USA [1]. Decisive factors jointly determining the efficiency of the electrochemical process are the reactions at the oxidizing anode as well as at the hydrogen evolving cathode. In two inspiring experimental studies [2–3], Subbaraman et al. reported enhanced hydrogen evolution activity in water splitting by tailoring TM(OH)_2_-Pt electro-catalyst/electrode assemblies, where TM represents Mn^2+^, Fe^2+^, Co^2+^ and Ni^2+^. The role of these hydroxides was to catalyze water dissociation. In this context, the objective of the present study is to contribute a novel descriptor for the electro-catalytic hydrogen evolution reaction (HER). It offers a complementary perspective on a recent study addressing the oxidation of zirconium alloys by water [4–5]. The overall reaction

**Figure 1:**
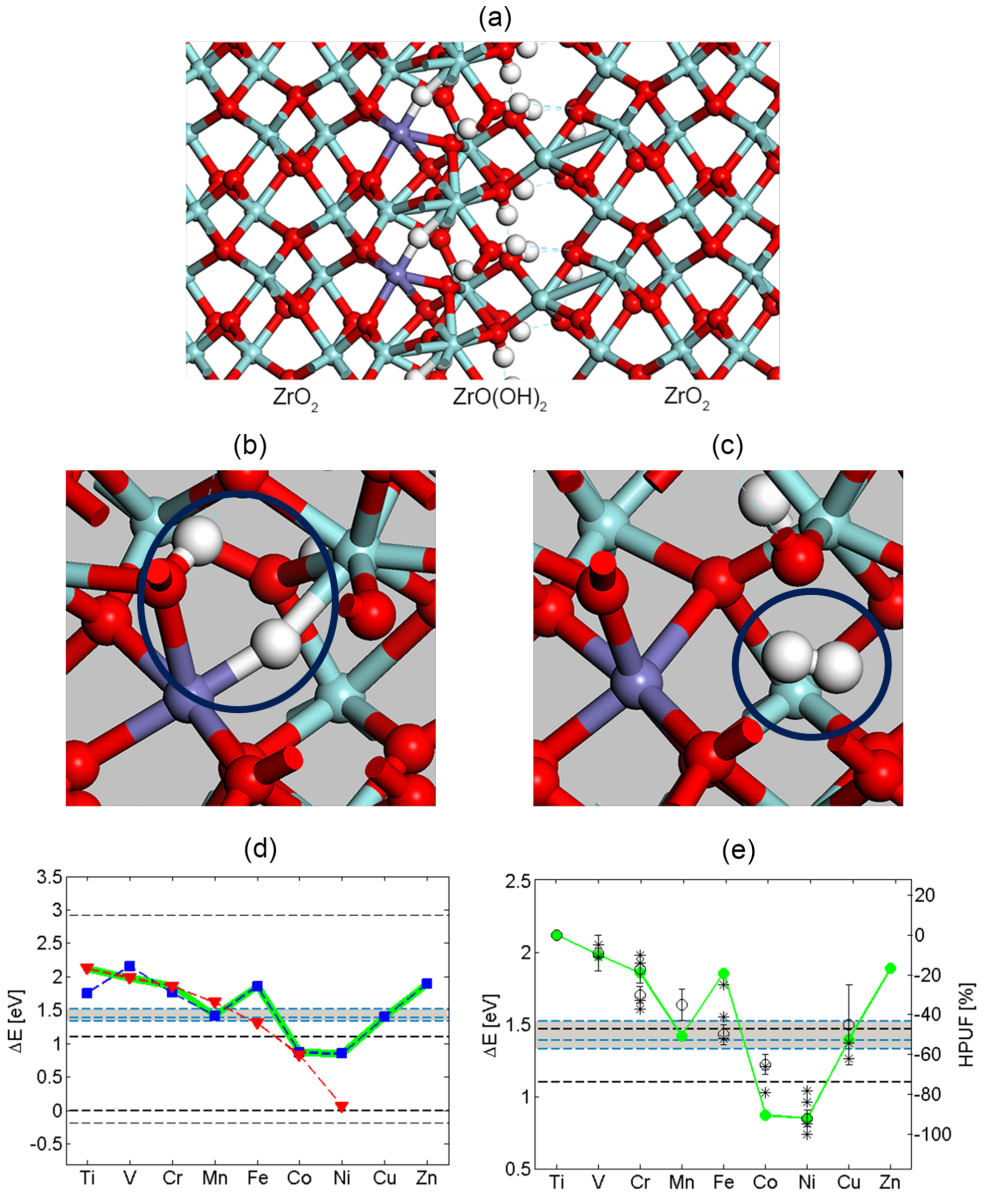
(a) Representative structure for a model of a hydroxylated inter-grain interface comprising ZrO(OH)_2_ on ZrO_2_. Here, this interface is decorated with Fe ions with the oxidation state +2. Oxygen is represented as red, zirconium as light blue, iron as purple, and hydrogen as white. (b) One hydride ion and one hydroxide moiety prior to the hydride-proton recombination to form H_2_ is displayed, reactant in Equation 6. (c) The product in Equation 6 is displayed, including M^X^ coordination to the additional oxygen ion replacing the hydride ion and the released grain boundary H_2_. (d) Hydride-proton recombination energies for H_2_ release into said interface (dashed black line at 1.1 eV), enthalpy change for H_2_ release at ambient pressure (dashed black line at 0 eV), and corresponding Gibbs energy change (dashed black line at −0.2 eV). TM^2+^ blue, TM^3+^ red, weighted average green. (e) Comparison of theoretical data (green) and experimental HPUF data (black); * from [7] and ^o^ from [8]. The theoretical data is a weighted average between TM^2+^ and TM^3+^. The black dashed line is HPUF in pure ZrO_2_ from [7]. The blue dashed line corresponds to HE from Zr^4+^ hydride at GB with Na^+^, Ca^2+^ and Sc^3+^ spectator. Sc^3+^ corresponds to the top line, Na^+^ to the middle line and Ca^2+^ to the bottom line.

[1]
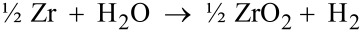


is taken to occur by water utilizing hydrolysis to penetrate the oxide scale along hydroxylated grain boundaries, see Figure 1a,

[2]
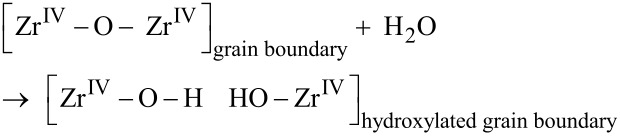


These hydroxide ions subsequently react with transition metal decorated sites (see Figure 1a) and zirconium metal to produce ZrO_2_ in conjunction with transient transition metal associated hydride-proton (hydroxide) pairs (see Figure 1b) to restore the ZrO_2_ grain boundary according to

[3]
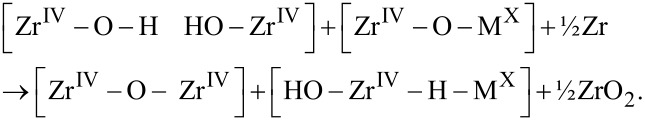


This can be subdivided into an anode process

[4]
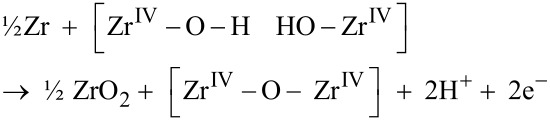


where the [Zr^IV^–O–Zr^IV^] oxide grain boundary is recovered, and a cathode process

[5]
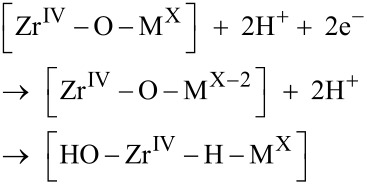


is employed to decide the oxidation state X. The subsequent chemical drive for H_2_ release into the confining grain boundary determines M and recovers the [Zr^IV^–O–M^X^] site (cf. Figure 1c)

[6]
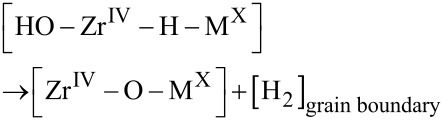


Indeed, Equation 6 was found to be decisive for the fraction of hydrogen atoms not forming H_2_ but being absorbed in the Zr alloy according to

[7]
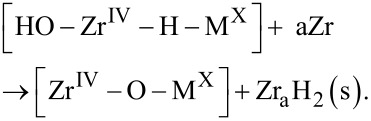


For completeness, a 1.1 eV/H_2_ drive to release H_2_ from the confining grain boundary was computed according to

[8]
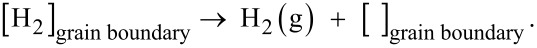


Utilization of the hydride-proton recombination channel (see Figure 1b), the correlation between the computed reaction energies for the HER, Equation 6, and the experimental hydrogen pick-up fractions (HPUF), i.e., the fraction of the hydrogen, which does not undergo hydrogen evolution but are instead picked-up by the alloy during zirconium oxidation by water, leads to a model as displayed in Figure 1e. It is noteworthy, that the energetics for the chemical reaction step in Equation 6 offers a measure of the confinement-dependent cathodic over-potential for the HER along the reaction channel (Equations 2–6). The relevance of the reversible hydride-proton recombination reaction (Equation 6) has recently been proposed in case of a nickel electro-catalyst supported by seven-membered cyclic diphosphine ligands containing one pendant amine, with the Ni supporting the hydride and the amine providing the proton in the hydride-proton recombination reaction [6].

## Results and Discussion

In the following we introduce and employ the notion of "confinement effect" as a steric Pauli repulsion type interaction between H_2_ and a hydroxylated interface (see Figure 1c) upon hydride-proton recombination. First, we employ this notion in the context of hydride-proton recombination reactions to demonstrate how it decides which oxidation state X of metal ion M minimizes the overpotential for the HER, as quantified by the reaction Equation 6 (cf. Figure 1d,e). Second, it is shown how the emerging understanding is naturally extended to include electro-catalysts for HER under ambient conditions.

### Impact of confinement on HER during zirconium oxidation by water

**Figure 2:**
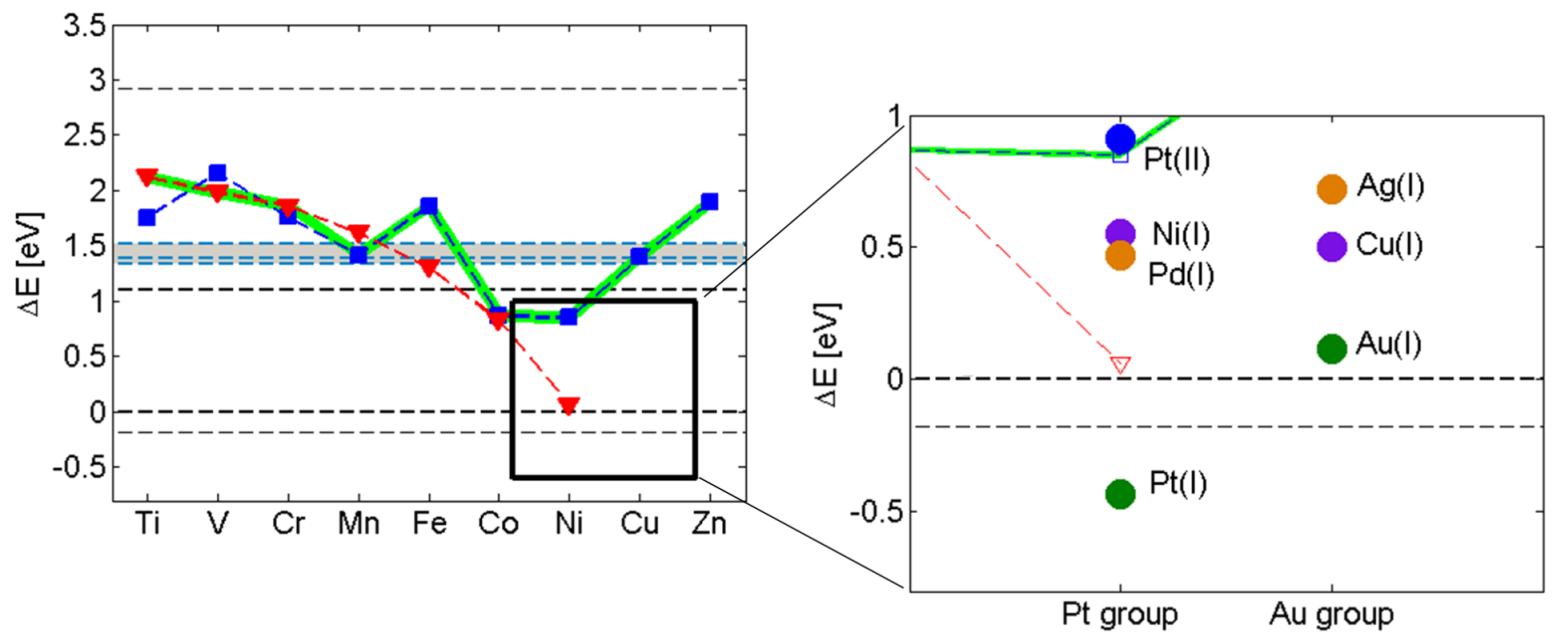
The diagram on the left is identical to Figure 1d. The enlarged region exposes the overpotentials for the elements in the Pt and Au groups. Note that Pt^+^ associated hydride displays a negative overpotential implying that it is more stable than the H_2_(g) asymptote (lower dashed line).

To investigate the confinement effect on the HER, we consider the zirconium oxidation by water (see Figure 1d). The difference between the two horizontal lines at 2.9 eV and at −0.2 eV corresponds to the 3.1 eV/H_2_ [9] thermodynamic drive for HER in case of Zr oxidation by water under ambient conditions (Figure 2). The line at 0 eV represents the energy of a free H_2_ molecule at 0 K. The line at 1.1 eV represents the energy cost at 0 K for bringing a free H_2_ molecule into the confinement represented by Figure 1c. The line at −0.2 eV is owing to the increase in entropy when a water molecules H_2_O(l) is consumed (−70.0 Jmol^−1^K^−1^ [10]) and a H_2_(g) molecule is formed (130.7 Jmol^−1^K^−1^ [10]) at 298 K and 100 kPa (compare Equation 1), while neglecting changes in entropy in Zr upon oxidation.

Thus, a perfect electro-catalyst would exhibit an enthalpy change of 0 eV for the HER under ambient conditions. Moreover, it is inferred that a perfect electro-catalyst, which passes the HER into this hydroxylated interface via Equation 6 prior to the subsequent H_2_ release under ambient conditions, must display 1.3 eV/H_2_ overpotential, i.e., (1.1 − (−0.2)) eV/H_2_. Equivalently, in case of the HER into the interface, any residual drive towards H_2_ formation relative to the line at 1.1 eV/H_2_ corresponds to a local overpotential for the HER into the confining interface. A correlation emerges between a greater overpotential and a lower hydrogen pick-up fraction (HPUF, see Figure 1d and 1e). Thus the well-known effect of Ni^2+^ to cause detrimental hydrogen pick-up was explained by its reluctance to release H_2_ into the hydroxylated internal inter-grain interface [4–5]. From the overall agreement between reaction energies for Equation 6 and the experimentally reported HPUF’s, it was concluded that "anti-catalysts" are preferred in order to mitigate the HPUF. In case of zirconium oxidation by water, these "anti-catalysts" are ions, which conserve significant fractions of the drive for hydrogen evolution by forming highly reactive metastable hydrides. These species are contrasted by Co^2+^ and Ni^2+^, which catalyze the HER when H_2_ is released into the highly constraining interface (see Figures 1d and 1e).

A stability check on the semi-quantitative validity of the model was offered by a comparison of the experimental 44% hydrogen pick-up fraction of pure zirconium (corresponding to the black horizontal dashed line at 1.5 eV/H_2_) and model calculations for the hydride-proton recombination energetics employing the inert Na^+^, Ca^2+^, and Sc^3+^ as "dummy" ions in the positions of the transition metal ions (see the three blue horizontal dashed lines in Figure 1e).

### On HER at ambient conditions – a consistency check

**Figure 3:**
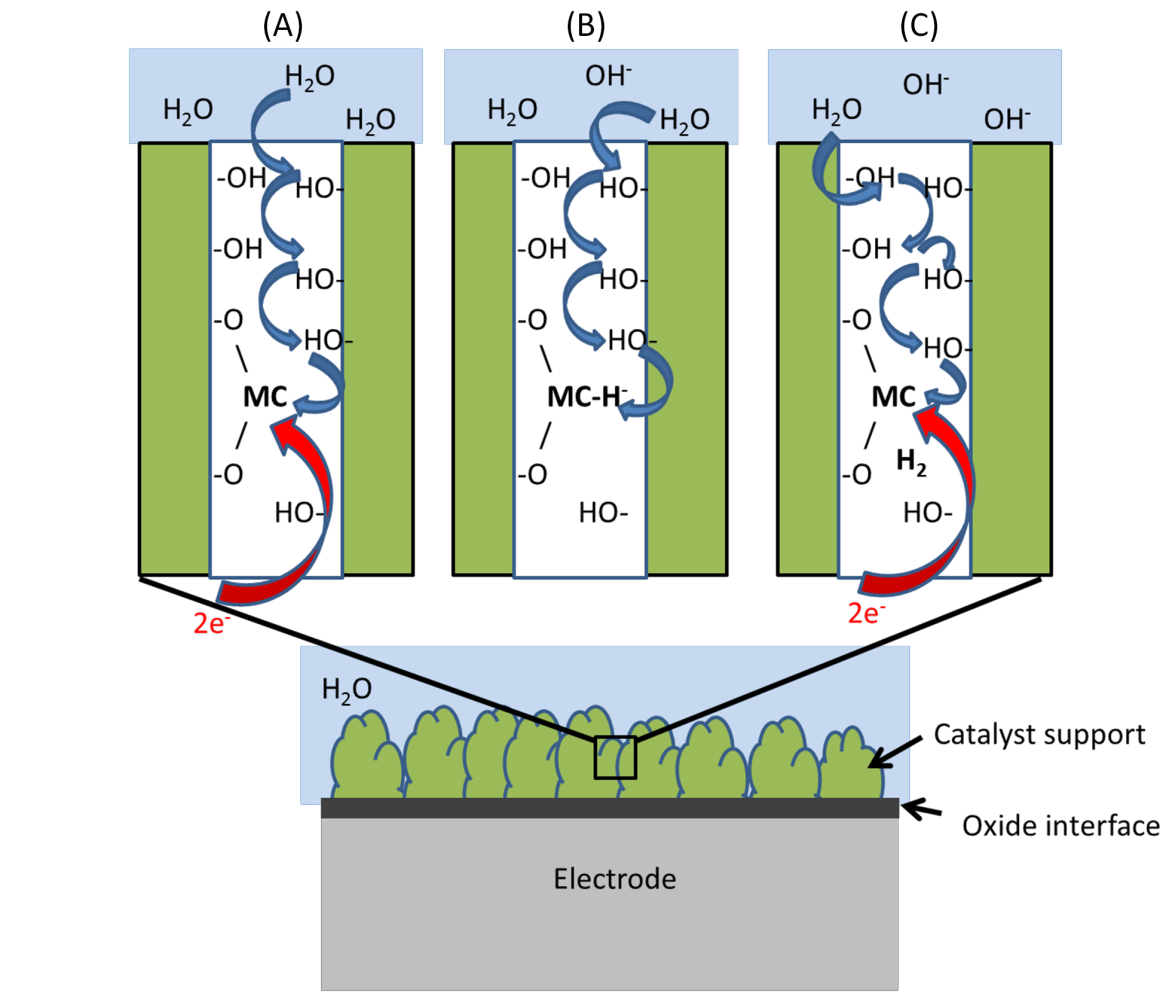
HER at electro-catalyst/electrode assembly. (A) Coalescence of proton and electrons to form the metal catalyst (MC) associated hydride, (B) Hydride-proton recombination to form H_2_ at the interface. (C) The step between panel B and panel C comprises the HER following the hydride-proton recombination step.

According to the above understanding, which ions constitute viable electro-catalysts in the absence of confinement or at atmospheric pressure? In as much as the drive for HER comprises the relaxation of the resulting oxy-hydroxy ions coordinating the transition metal ion [4], it is suggested that besides being able to form the hydride intermediate, metals with low oxidation states and large ionic radii should be considered in order to minimize their affinities to the oxide surrounding. This characterization clearly points to the noble metal ions as candidates for electro-catalysts. Additional requirements for any successful electro-catalyst include sufficient electron conductivity of the oxide matrix supporting the catalyst as well as electric contact to the electrode itself. Finally, the "water dissociation catalysis" put forward by Subbaraman et al. [2–3] is used to infer that hydroxylated interfaces provide natural channels for proton transport to the oxy-hydroxide supported electro-catalytic site. A schematic representation of this understanding of the electrode/electro-catalyst assembly is provided in Figure 3.

Employing the above described hydroxylated inter-grain interface model as a generic supporting matrix for the eletrocatalytic process, we evaluate the energetics for the hydride-proton recombination reaction and arrive at a possible descriptor for the HER, which is also applicable under ambient conditions. This facilitates a procedure for the screening among candidate electro-catalysts.

Indeed, in Figure 2, a descending staircase-like curve for electro-catalysts is arrived at for the reaction energy corresponding to Equation 6. Starting at the hydroxylated zirconia inter-grain confinement, where Co^2+^ and Ni^2+^ are the obvious candidate catalysts, we approach the ambient conditions step by step by considering the embedded Ag^+^, Ni^+^, Cu^+^, Pd^+^, Au^+^ and eventually Pt^+^. The Sabatier principle applies in two ways. Firstly, X in M^X^ can be made to satisfy the requirement that the reactant in Equation 6 forms spontaneously [4–5]. Secondly, the environment confining H_2_ in Equation 6 can be tuned to equalize the stabilities of reactants and products, so that any drive to release H_2_ into a confinement is balanced owing to the replacement of the three-centered hydride by an oxy-bridge (Equation 6). It is gratifying to find that the oxy-hydroxide supported Au^+^ and Pt^+^ sites become preferred only when approaching ambient conditions, as oxides of noble metals are generally unstable, a property which is often associated with their softness. Consequently, the drive to replace the hydride ion by an oxygen ion is weak, and hence the H_2_ release is expected to require a loose confinement for these systems. This is in contrast to harder ions, which form more stable oxides. Interestingly, in case of Pt^+^, the hydride comes out more stable than the H_2_(g) limit. This implies that the embedded Pt^+^ site could constitute an efficient absorber of H_2_ in the gas phase under ambient conditions - a purely chemical property. The semi-quantitative nature of the methodology does not allow for precise predictions of absolute numbers (see horizontal "error bar" in Figure 1e). However, it may be that the overpotentials reported for the Pt-based catalysts are related to the coverage dependence of the electrochemical decomposition of the Pt^+^ associated hydride compound. Detailed properties of the embedding materials (e.g., electron conductivity) could cause the additional variations of the overpotential observed by Subbaraman et al. [2].

Interestingly, +2 is not considered a relevant oxidation state in case of Pt under ambient conditions for the electro-catalytic reaction path involving the hydride-proton recombination reaction (see Figure 2). This result is due to the strong binding of +2 to the oxy-hydroxide ligands upon H_2_ release, violating Sabatiers principle.

In conclusion, the present approach offers a complementary computational strategy to rank catalysts for HER from water splitting. The complex modelling of heterogeneous HER electro-catalysis at the interface between composite catalyst/support and a water based electrolyte is subdivided into a chemical oxide hydrolysis step (Equation 2), an electro-chemical redox step (Equation 3, Equation 4 and Equation 5), followed by the chemical hydride-proton recombination step (Equation 6). This conceptual deconstruction aims for supporting the prediction of novel approaches to improve on existing electro-catalyst/electrode assemblies. Thus, the design of the aqueous electrolyte/substrate system impacts only the hydrolysis step (Equation 2). The oxidation state X of M^X^ is decided by Equation 5, while the choice of supported HER catalyst M^X^ is determined according to Equation 6 by the confinement effect in conjunction with Sabatier's principle.

For the HER step, a recently proposed alternative to the Volmer–Heyrovsky mechanism was employed [4–5]. Rather than electron-proton discharge over an M–H moiety resulting in the conversion of 2H into H_2_, the HER investigated here results from a hydride-proton recombination reaction. While the protons constitute hydroxides in Equation 6, which is non-significant due to their ubiquity in aqueous media, an observation of three-center hydride intermediates is the sought-after "smoking-gun" evidence for the proposed mechanism.

### Computational details

The Perdew–Burke–Ernzerhof generalized gradient approximation PBE GGA [11] as implemented in the DMOL3 engine [12–13] in the Material Studios program package [14] was employed in conjunction with a double-ζ numerical basis set with an extra polarization function on each heavy atom and a p-function on each hydrogen atom. Systematic spin polarized calculations were performed. A 4 × 4 × 1 k-point set for sampling the Brillouin zone was compared to a 2 × 2 × 1 k-point set, and the latter was found to suffice. In order to reduce the computational effort, inert electron shells were described effectively by means of the semi-core pseudopotentials. Zero-point corrected free energies were compared to non-corrected reaction energies and the differences were deemed negligible.

The grain boundary model (cf. Figure 1a) was constructed by inserting one unit cell of monoclinic ZrO(OH)_2_ (5.4 Å × 10 Å × 5.4 Å) in between two supercells of monoclinic ZrO_2_ (5.4 Å × 10.8 Å × 5.4 Å), where the unit cell doubling is in the b-direction. The stability of the model has been extensively investigated, including full geometry optimization, when arriving at the foundation of [4]. The choice of the grain boundary model is far from unique. Here, it is the success in reproducing the experimental volcano shape curve (cf. Figure 1d) which renders the present investigation meaningful.

The grain boundary model employed to evaluate the reaction energy of Equation 6 was subjected to periodic boundary conditions, where the studied super-cell contained approximately 50 atoms. The number of hydrogen atoms, i.e., hydroxides and hydride, varied. This was because the oxidation states of the transition metal ions were controlled by adding (removing) hydrogen atoms to (from) the super-cell. This way, neutral super-cells were employed in all cases. When evaluating Equation 6, all bond distances and bond angles associated with atoms in the super-cell were optimized, while the super-cell dimensions were kept constant.
